# Predicting experimental success: a retrospective case-control study using the rat intraluminal thread model of stroke

**DOI:** 10.1242/dmm.044651

**Published:** 2020-12-29

**Authors:** Lisa Liebenstund, Mark Coburn, Christina Fitzner, Antje Willuweit, Karl-Josef Langen, Jingjin Liu, Michael Veldeman, Anke Höllig

**Affiliations:** 1 Department of Anesthesiology, University Hospital Aachen, Rheinisch-Westfälische Technische Hochschule (RWTH) Aachen University, D-52074 Aachen, Germany; 2 3CARE, Cardiovascular Critical Care & Anesthesia Research, University Hospital Aachen, RWTH Aachen University, D-52047 Aachen, Germany; 3 Institute of Neuroscience and Medicine, Medical Imaging Physics (INM-4), Forschungszentrum Jülich GmbH, D-52428 Jülich, Germany; 4 Department of Nuclear Medicine, University Hospital Aachen, RWTH Aachen University, D-52047 Aachen, Germany; 5 Department of Neurosurgery, University Hospital Aachen, RWTH Aachen University, D-52047 Aachen, Germany

**Keywords:** tMCAO, Rat model, Stroke animal model, Peri-interventional monitoring, Experimental quality assurance

## Abstract

The poor translational success rate of preclinical stroke research may partly be due to inaccurate modelling of the disease. We provide data on transient middle cerebral artery occlusion (tMCAO) experiments, including detailed intraoperative monitoring to elaborate predictors indicating experimental success (ischemia without occurrence of confounding pathologies). The tMCAO monitoring data (bilateral cerebral blood flow, CBF; heart rate, HR; and mean arterial pressure, MAP) of 16 animals with an ‘ideal’ outcome (MCA-ischemia), and 48 animals with additional or other pathologies (subdural haematoma or subarachnoid haemorrhage), were checked for their prognostic performance (receiver operating characteristic curve and area under the curve, AUC). Animals showing a decrease in the contralateral CBF at the time of MCA occlusion suffered from unintended pathologies. Implementation of baseline MAP, in addition to baseline HR (AUC, 0.83, 95% c.i. 0.68 to 0.97), increased prognostic relevance (AUC, 0.89, 95% c.i. 0.79 to 0.98). Prediction performance improved when two additional predictors referring to differences in left and right CBF were considered (AUC, 1.00, 95% c.i. 1.0 to 1.0). Our data underline the importance of peri-interventional monitoring to verify a successful experimental performance in order to ensure a disease model as homogeneous as possible.

## Abstract (precis)

**Summary:** An assessment of the importance of peri-interventional monitoring to verify a successful experimental performance in order to ensure a disease model that is as homogeneous as possible.

## INTRODUCTION

Despite extensive clinical and experimental research in the field of ischemic stroke, findings rarely take the decisive step from bench to bedside (Endres et al., 2008). Basically, there are various pitfalls and problems that have to be addressed in order to produce clinically relevant and reproducible results (Tymianski, 2015). One aspect is the documentation and reporting of studies, including results and limitations. Clinical scientists started to implement clear and structured reporting guidelines in order to provide transparent and complete reporting of randomized clinical trials, resulting in the publication of the Standardized Reporting of Trials statement in 1994, which has been developed further in 1996 (Consolidated Standards of Reporting Trials Statement), as a result of the merger with the simultaneously developed Asilomar Guideline (by the Asilomar Working Group on Recommendations for Reporting of Clinical Trials in the Biomedical Literature; Andrew et al., 1994; Fackler et al., 1994; Begg et al., 1996). In line with this effort to increase the transparency and improve the quality of studies with regards to preclinical research, several calls (Landis et al., 2012) and recommendations, such as the Stroke Therapy Academic Industry Roundtable recommendations [initially published in 1999 (STAIR, 1999), with updates in 2009 (Fisher et al., 2009) and 2019 (Savitz et al., 2019)] and the Animal Research: Reporting of *In Vivo* Experiments (ARRIVE) criteria (published in 2010; Kilkenny et al., 2010) have been published. The Ischaemia Models: Procedural Refinements Of in Vivo Experiments (IMPROVE) guidelines also provide a detailed guide for the performance of ischemia models (Percie du Sert et al., 2017).

Another aspect is the experimental methodology itself – the most common model in preclinical stroke research is the middle cerebral artery occlusion (MCAO) model established by Koizumi et al. (1986) and later modified by Longa et al. (1989). It is a legitimate objection to investigate whether the common animal models actually reflect the disease exhaustively, whether statistical instruments (as effect sizes) are used properly, whether endpoints are chosen reasonably (before the start of the study), and whether the complexity of the pathology is actually considered (Endres et al., 2008; Sughrue et al., 2010; Hossmann, 2012; Landis et al., 2012; Mergenthaler and Meisel, 2012). However, from a technical point of view, one also has to consider whether the model is applied correctly or whether there might be scope for improvement – reproducibility of preclinical data is a major issue (Prinz et al., 2011; Llovera et al., 2015).

Unfortunately, owing to heterogeneous experimental setups, studies using the MCAO model bear the risk of inconsistent outcomes and limited reproducibility (Braeuninger and Kleinschnitz, 2009). The extent of the ischemic damage varies highly due to the variable cerebrovascular anatomy of different rat strains (Oliff et al., 1995; Bardutzky et al., 2005; Walberer et al., 2006), different types of filaments (Bouley et al., 2007; Zhao et al., 2008) or anaesthesia (Zausinger et al., 2002; Zhao et al., 2008). Furthermore, the methods applied to verify correct MCAO (resulting in cerebral ischemia) vary highly: during the intervention, indirect methods are available to ascertain correct MCAO, e.g. via measurement of cerebral blood flow (CBF; Engelhorn et al., 2005; Bleilevens et al., 2013) or, with certain limitations, electroencephalography (Schmid-Elsaesser et al., 1998; Hungerhuber et al., 2006); and afterwards, histological staining of brain sections (Kramer et al., 2010; Rousselet et al., 2012) or MRI (Iskander et al., 2013; Trotman-Lucas et al., 2017) provide evidence for ischemia. The impact of ischemia, to some degree, can be described by neurological deficits using neurological severity assessment scores (Bederson et al., 1986; Garcia et al., 1995) or behavioural tests (Schallert, 2006; Encarnacion et al., 2011). However, one major drawback of the intraluminal method is the occurrence of model-immanent confounding pathologies and complications, such as incomplete MCAO (Zausinger et al., 2002), early, late or missing reperfusion (Livnat et al., 2010), and subarachnoid haemorrhage (Longa et al., 1989; Schmid-Elsaesser et al., 1998). With particular regard to time- and labour-consuming long-term experiments, a reliable and prompt surveillance of the desired vessel occlusion excluding any secondary damages is of great importance because MRI is not always available or feasible, and the additional lesions at the time of sacrifice may no longer be recognizable and therefore results may be biased.

We present data on a selection of MCAO experiments, including detailed monitoring of bilateral CBF, heart rate (HR) and systolic/diastolic pressure, and mean artery blood pressure. The aim of our secondary analysis is the identification of predictors and their optimal combination to anticipate a successful induction of ischemic stroke, thus the early detection of confounding pathologies, such as subarachnoid haemorrhage (SAH) and subdural hematoma (SDH).

## RESULTS

### Comparison of outcome groups

**Fig. 1.**
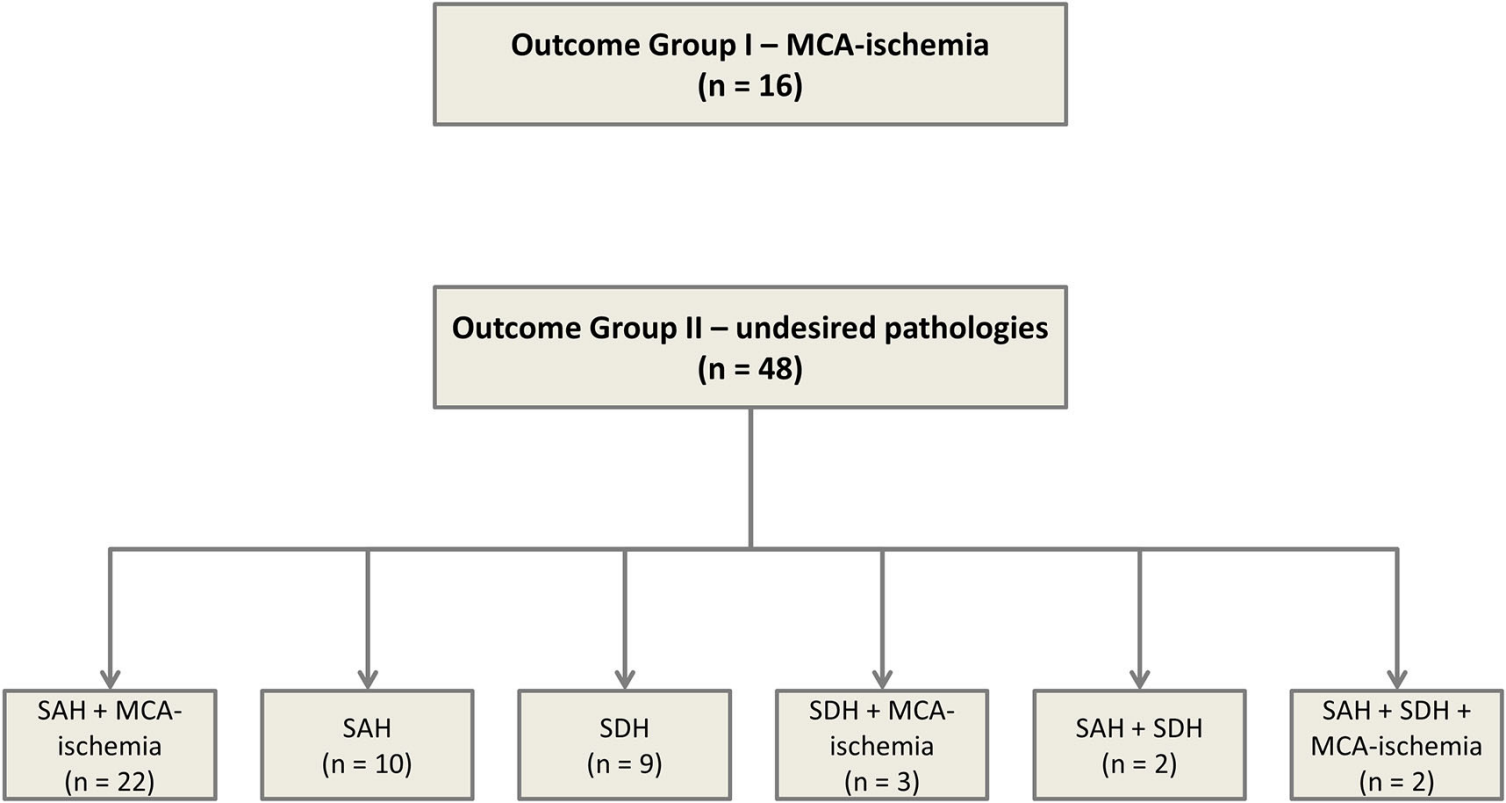
**Flowchart of animals enrolled in the study, including specific pathologies detected.**

In order to identify predictors indicating undesired results at an early stage of the experiment, we compared two groups of animals representing the extremes (‘ideal’ versus ‘undesired’ outcome): outcome group I incorporated 16 animals with MCA-ischemia, whereas outcome group II was composed of 48 animals presenting ‘undesired’ results, such as SAH or SDH. The specific diagnoses are presented in Fig. 1.

**Fig. 2.**
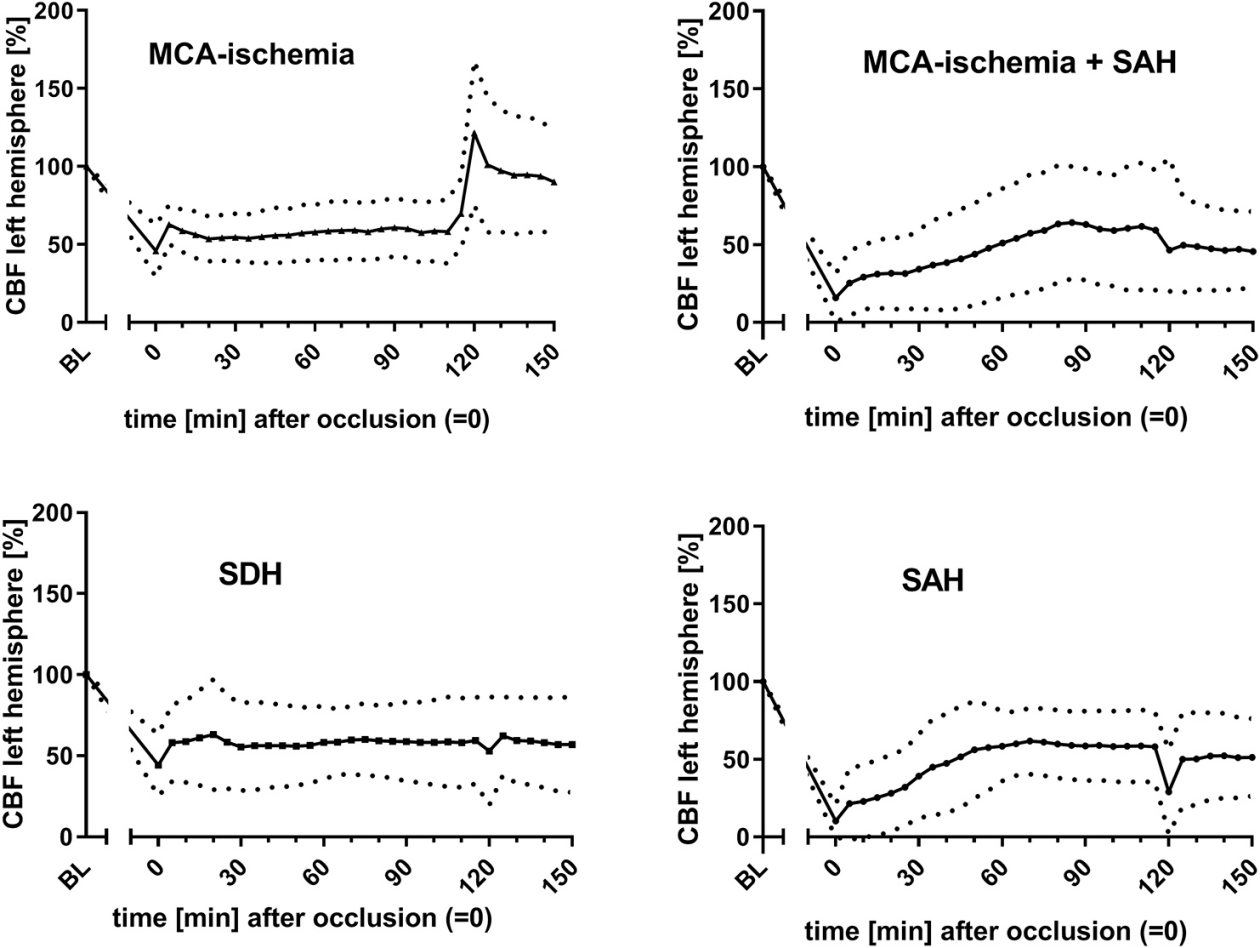
**Courses of lCBF dependent on outcome.** Graphs show a specific pattern of lCBF when MCA-ischemia was achieved; whereas the drop of lCBF in animals with SAH was more distinct, lacking the increase indicating reperfusion. Data are mean±s.d. (mean is represented as continuous lines and s.d. is represented as dotted lines). *x*-axis, time (0 is set for occlusion time); *y*-axis, CBF of left hemisphere.

**Fig. 3.**
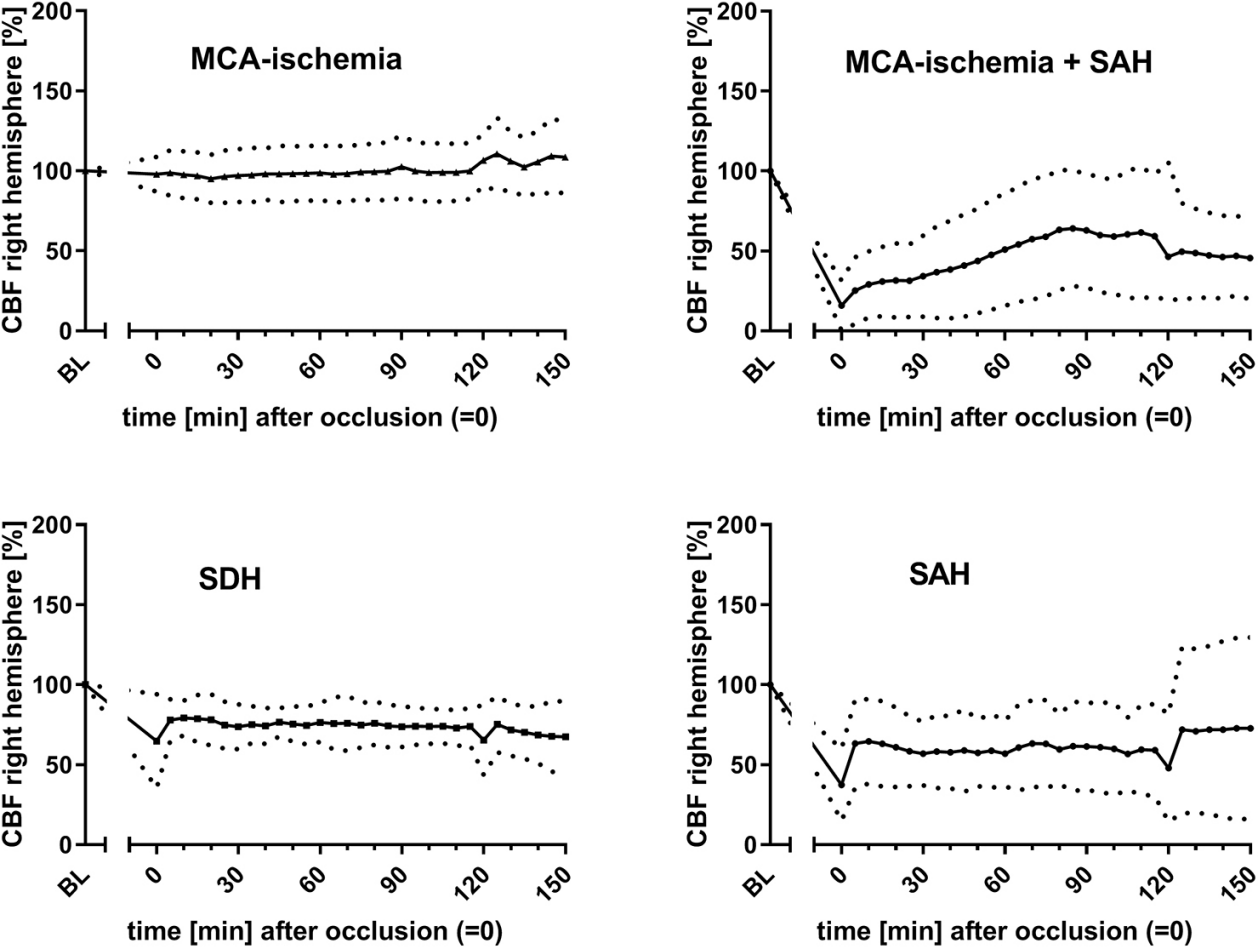
**Courses of rCBF dependent on outcome.** Graphs show a stable rCBF pattern for animals with MCA-ischemia. Data are mean±s.d. (mean is represented as continuous lines and s.d. is represented as dotted lines). *x*-axis, time (0 is set for occlusion time); *y*-axis, CBF of left hemisphere.

**Fig. 4.**
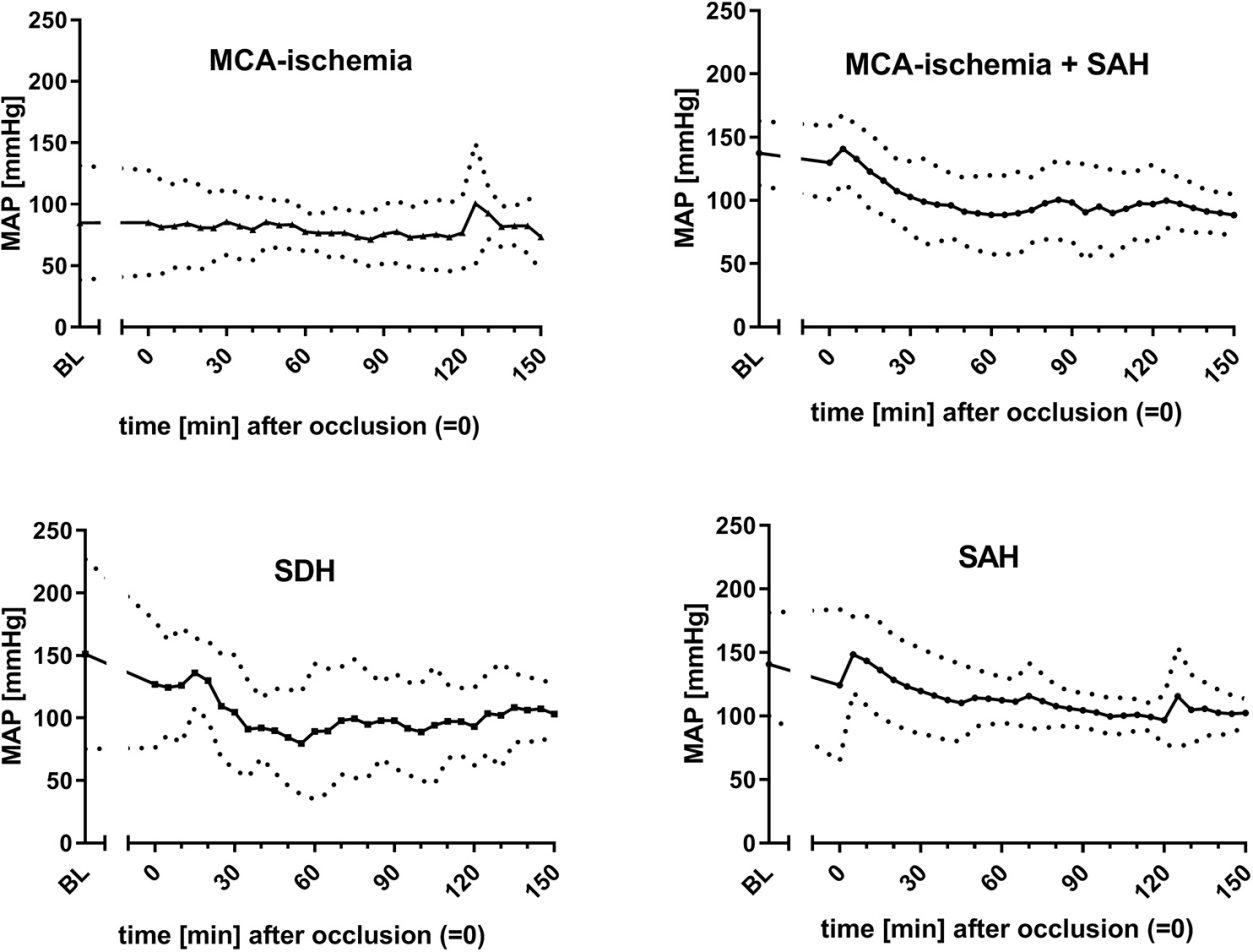
**Courses of MAP dependent on outcome.** Graphs show a lower baseline MAP in animals with MCA-ischemia compared to animals with other pathologies. Data are mean±s.d. (mean is represented as continuous lines and s.d. is represented as dotted lines). *x*-axis, time (0 is set for occlusion time); *y*-axis, MAP.

**Fig. 5.**
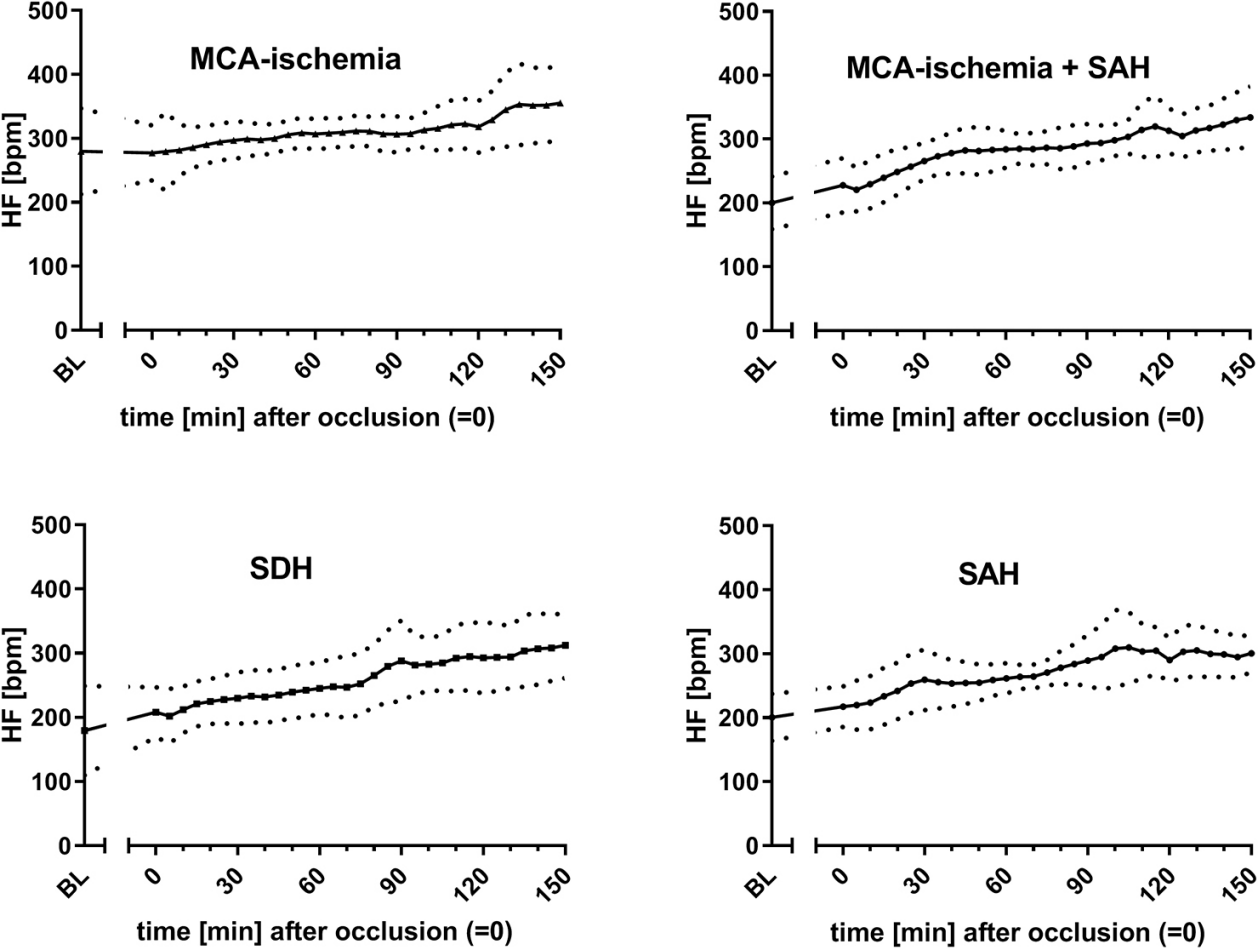
**Courses of HF dependent on outcome.** Data are mean±s.d. (mean is represented as continuous lines and s.d. is represented as dotted lines). *x*-axis, time (0 is set for occlusion time); *y*-axis, HF.

For outcome group I, the infarct volume was assessed. A median infarct volume of 30.5% (±13.5%) was found. The most common findings among the ‘undesired’ results (outcome group II) were SAH (*n*=9), SDH (*n*=7) and SAH additional to MCA-ischemia (*n*=22). The courses of bilateral CBF (Figs 2, 3), MAP (Fig. 4) and heart frequency (HF) (Fig. 5), according to the specific diagnoses, are depicted below. Results of blood gas analyses have been partially published previously (Liu et al., 2019).

### Description and correlation of single predictors

The courses of the CBF showed particularly distinct patterns according to the final diagnoses: in outcome group I (MCA-ischemia), a sharp decrease of left CBF (lCBF; up to 75%) indicated occlusion of MCA, whereas right CBF (rCBF) remained relatively stable (Figs 2, 3). When the filament was withdrawn after 120 min lCBF increased immediately (in some cases even above the baseline) and was then followed by a gradual decrease ipsilaterally. Right CBF showed zero or only a minor increase after withdrawal. A steep decrease of the rCBF was seen when ischemia was accompanied by SAH or SAH alone was detected (Fig. 3).

**Fig. 6.**
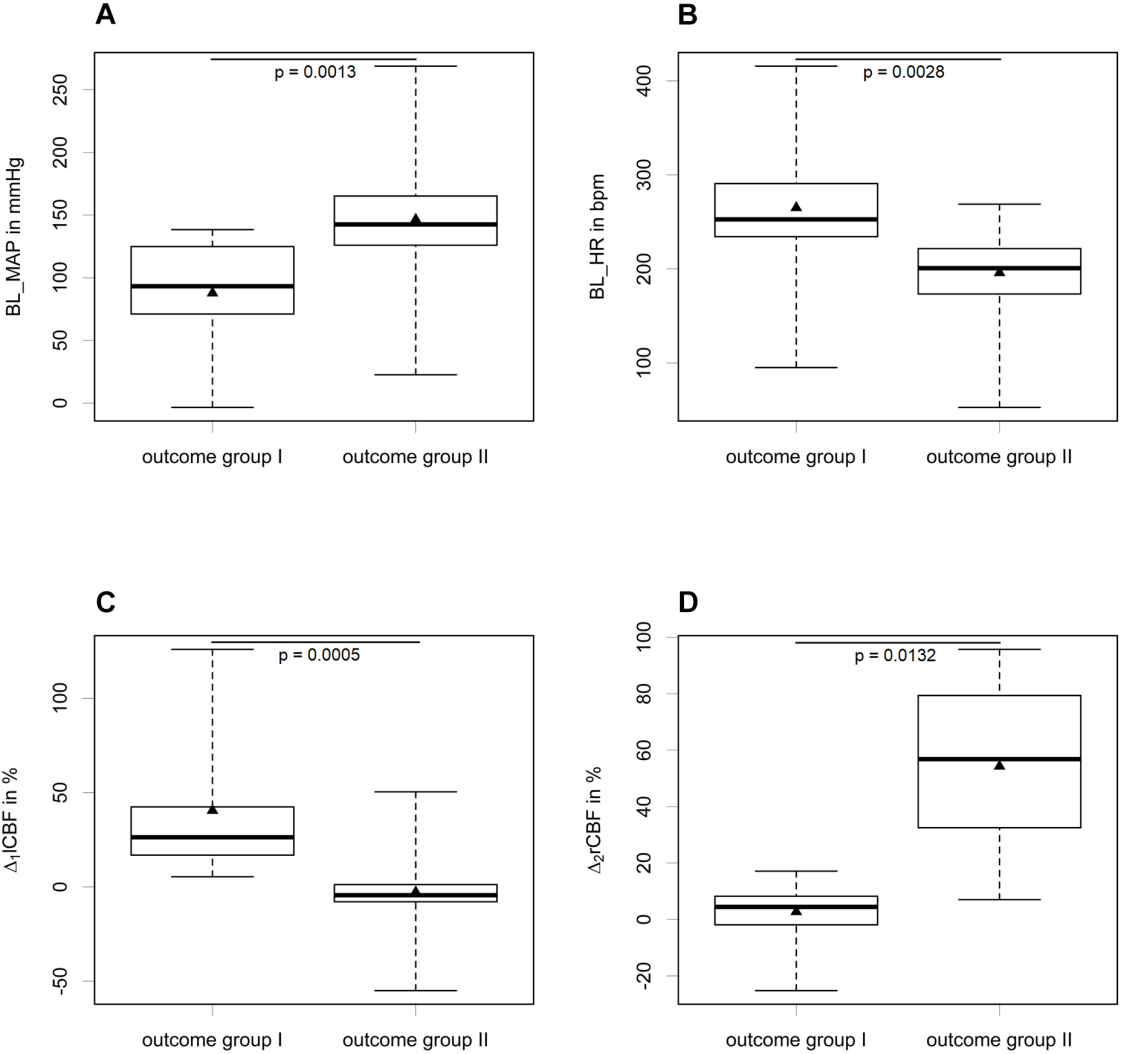
**Boxplots comparing predictor parameters between outcome groups I and II.** (A) Comparison of BL_MAP in mmHg between outcome groups I (*n*=16) and II (*n*=48). (B) Comparison of BL_HR in bpm between outcome groups I (*n*=16) and II (*n*=48). (C) Comparison of predictor variable Δ_1_lCBF [difference between mean lCBF before (t0 to<t120) and after (t120 to t150) occlusion] (as a percentage) between outcome groups I (*n*=15) and II (*n*=44). (D) Comparison of predictor variable Δ_2_rCBF (difference between rCBF at baseline and t0) (as a percentage) between outcome groups I (*n*=15) and II (*n*=45). For all parameters, outcome groups I and II differ significantly (BL_MAP, *P*=0.0013; BL_HR, *P*=0.0028; Δ_1_lCBF, *P*=0.0005; Δ_2_rCBF: *P*=0.0132). Mean values are indicated by triangles. The boxes represent quartiles 1 and 3, and the whiskers represent minimum and maximum values. Statistical significance was determined by a Mann–Whitney U-test.

Baseline HR [BL_HR; outcome group I, 263.3±76.3 beats per minute (bpm); outcome group II, 198±42.8 bpm; *P*=0.0028] and baseline MAP (BL_MAP; outcome group I, 90.4±44.5 mmHg; outcome group II, 144.2±38.8 mmHg; *P*=0.0013) both differed significantly between the two outcome groups (univariate logistic regression; Fig. 6A,B). Additionally, Δ_1_lCBF (outcome group I, 40.5±37.4%; group II, −2.8±15%; *P*=0.0005) and Δ_2_rCBF (outcome group I, 2.7±10.6%; outcome group II, 54.9±26.9%; *P*=0.0132) were selected as specific predictor variables (see Fig. 6C,D). The initial weight of both outcome groups did not differ significantly (outcome group I, 349.3±36.7 g; outcome group II, 363.7±42.8 g; *P*=0.2348). Moderate to strong correlations were found for BL_HR and Δ_2_rCBF (Pearson *r*=−0.40, 95% c.i. −0.59 to −0.16), and BL_HR and Δ_1_lCBF (*r*=0.34, 95% c.i. 0.09 to 0.55), whereas an inverse correlation was seen for BL_MAP and Δ_1_lCBF (*r*=−0.41, 95% c.i. −0.60 to −0.17) (Fig. S1).

### Prediction of confounding pathologies

Using only single predictors, BL_HR yielded the lowest area under the curve (AUC) of 0.83 (95% c.i. 0.68 to 0.97), whereas the AUC was slightly higher when BL_MAP was used (0.84; 95% c.i. 0.73 to 0.95). The AUC of Δ_1_lCBF and Δ_2_rCBF proved to be even higher at 0.95 (95% c.i. 0.89 to 1.00) and 0.97 (95% c.i. 0.93 to 1.00), respectively. Owing to low AUC values, baseline systolic (BL_SYS), diastolic blood pressure (BL_DIAS), Δ_3_lCBF and mean_rCBF were excluded as predictors from further analysis (Tables S1, S2). Adding a second parameter (BL_HR and BL_MAP) to the model increased the AUC (0.89, 95% c.i. 0.79 to 0.98). The average area under the receiver operating characteristic curve (ROC) using BL_HR+Δ_1_lCBF+Δ_2_rCBF was 0.99 (95% c.i. 1.0 to 1.0). Prediction performance improved when all four predictors (BL_HR+BL_MAP+Δ_1_lCBF+Δ_2_rCBF) were taken into account (AUC 1.00, 95% c.i. 1.0 to 1.0), indicating perfect discrimination (for details of the prediction model see Table 1 and Table S3). Small Brier scores (close to zero) indicate better forecasts. In our model this score ranged from 0.13 using one predictor to 0.00 using four predictors. The entire statistics, including all performance measures, odds ratios and cross validation are shown in detail in the supplementary Materials and Methods.

**Table 1.**
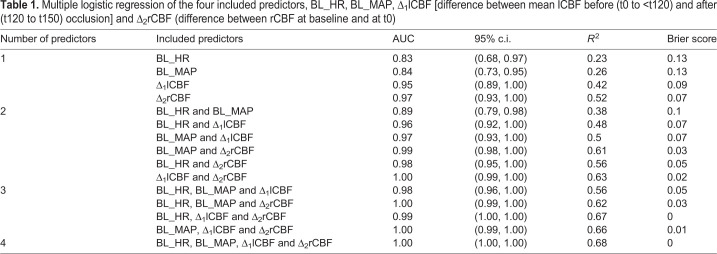
Multiple logistic regression of the four included predictors, BL_HR, BL_MAP, Δ_1_lCBF [difference between mean lCBF before (t0 to <t120) and after (t120 to t150) occlusion] and Δ_2_rCBF (difference between rCBF at baseline and at t0)

## DISCUSSION

Our data underline the importance of perioperative monitoring for the intraluminal thread model of stroke in rats – there is a clear predictive value of bilateral CBF, HR and MAP measurement with regards to undesired results, such as SAH or SDH. Bilateral CBF measurement offers simultaneous surveillance of both MCA-territories and, as far as our experience goes, clearly indicated the occurrence of SAH. Higher initial HR and low MAP at baseline were associated with the successful induction of MCA-ischemia.

### Bilateral CBF monitoring via laser Doppler flow

Yet in 1998, with regards to the MCAO model, Schmid-Elsaesser et al. (1998) reported the occurrence of SAH in up to 30% of the cases and stressed the importance of bilateral CBF measurement as a sensitive tool to identify SAH. However, there are still an enormous number of publications about experiments using the MCAO model without perioperative monitoring. Bearing in mind the ‘1026 experimental treatments in acute stroke’ from 2006 (O'Collins et al., 2006), it has to be mentioned that, from a methodological point of view, there is not only a lack of modelling risk factors (such as age) and confounding diseases (e.g. atherosclerosis) (Endres et al., 2008), but also an absence of a sophisticated periprocedural surveillance to assure experimental success, as undesired pathologies, with or without ischemia, may falsify the experimental result, particularly if unperceived. In contrast to the data presented by Woitzik and Schilling (2002), who advocated the use of a unilateral CBF measurement, our data did not allow a distinct discrimination of MCA-ischemia, ischemia plus SAH, SAH and SDH by analyzing lCBF exclusively, even though a partial recovery has been observed. Furthermore, by examining a relatively large cohort, we were able to detect various complications that all may be discriminated from MCA-ischemia by analyzing CBF bilaterally with an AUC of 1.0. Thus, by bilateral CBF measurement, a reliable control of the experimental results is granted, which is essential, particularly in the case of long-term experiments in which the chance to identify undesired pathologies, such as SAH macroscopically or histologically, diminishes by time. Strikingly, the results of a survey within the scientific community on ‘Phase III Preclinical Trials in Translational Stroke Research’ revealed that 38% of the participants do not attach an importance to the periprocedural CBF monitoring (Boltze et al., 2016). Thus, the authors conclude that a ‘broader awareness of the necessity to thoroughly control stroke induction’ is needed (Boltze et al., 2016).

### Blood pressure and HR monitoring

Basically, several guidelines recommend monitoring blood pressure closely (Liu et al., 2009; Howells et al., 2010). The successful induction of stroke in the intraluminal thread model has not been analyzed as a function of BL_MAP or BL_HR up to now. Our results suggest that the successful induction of ischemic strokes is more likely in the presence of lower preischemic MAP values, whereas undesired pathologies such as SAH or SDH occur more frequently when preischemic MAP values are higher.

The different BL_MAP and BL_HR values in the outcome groups might be a result of the inter-individual sensitivity of animals to anaesthetic and analgesic drugs (Davis, 2008). These variations can be attributed to differences in age, weight, genetic background of the strain (inbred or outbred), sex, age, body temperature, nutritional/health status (e.g. acid-base metabolism, haemodynamics and pulmonary function), circadian rhythm and endocrine factors (Avsaroglu et al., 2007; Davis, 2008).

Of note, the filament model generally shows a high incidence of reperfusion-associated parenchymal hematomas in spontaneously hypertensive rats (SHR) (Henning et al., 2008), which have been established to bring autonomic and cardiovascular effects of stroke into focus and, thus, mimic the pathophysiological human background more specifically. In spontaneously hypertensive animals, significantly more frequent vascular injury or haemorrhagic infarction after 3 h of transient MCAO compared to normotensive rats has been reported (Guan et al., 2011). This vascular vulnerability and pre-existing damage in animals with higher blood pressure, resulting in endothelial dysfunction, is a possible explanation for the detected susceptibility of the rats to unintended lesions (Yao and Nabika, 2012).

Blood pressure and HR naturally interact and, thus, in any case of hypotension a compensatory rise of HR can be detected (Groth et al., 2003). Therefore, the correlation of HR and MAP is obvious. The specific rise of HR may occur as a result of hypotension induced by anaesthesia, as individual reactions in response to analgesic and anaesthetic medications are common (Davis, 2008), and may explain the variability of physiological parameters.

### Limitations

Limitations of this study arise from its retrospective character: we processed selected data from an animal study in order to perform this subanalysis to compare the ‘extreme’ results (ideal versus undesired outcome). Observation times differed within the cohort, but no animals attributed to a long-term observation were included. Furthermore, the applied method of CBF measurement was biased by confounding effects such as moving artifacts and heterogeneous vascular supply. Blood gas analyses (usually at least three measurements during the procedure) were not available for all of the animals included. Furthermore, our results are limited to our specific setting (male Wistar rats, injectable anaesthetics, ventilated animals and transient occlusion of the MCA). Finally, we cannot propose a final prediction model yet, and further research and external validation is required.

### Translational approach and recommendations

Considering the enormous efforts and financial investments in preclinical stroke research, the translational power remains disappointing. Various aspects may contribute to the translational failure. There are fundamental concerns, such as whether the models used actually reflect the pathology adequately (Hossmann, 2012; Mergenthaler and Meisel, 2012), and whether the experimental methodology (including realistic estimation of effect sizes and sample size planning, as well as quality assurance and reporting) is considered responsibly (Braeuninger and Kleinschnitz, 2009; Sughrue et al., 2010; Landis et al., 2012; Llovera et al., 2015; Boltze et al., 2016). Beyond that, the predominant inclusion of young adult rats does not reflect the human epidemiology of ischemic stroke. The same issue applies for the health status of laboratory animals: there are several animal models resembling our diverse and ageing society that stroke research should not omit. Therefore, an important issue is the implementation of sex- and age-balanced animals (Endres et al., 2008; McCullough et al., 2014). Furthermore, the methodological quality of preclinical trials varies (Philip et al., 2009). However, the entire debate is also crosslinked with other aspects, such as science policy, funding strategies and scientific culture (acknowledging also negative trials and experimental failures) (Begley et al., 2015; Tymianski, 2015).

Our data confirm and support the concrete recommendations for practical application put forward by the IMPROVE guidelines and several other guidelines (Liu et al., 2009; Howells et al., 2010; Percie du Sert et al., 2017). Among other things, the highest possible reproducibility of a study is achieved by implementing various intraoperative monitoring procedures (Percie du Sert et al., 2017). Based on our data, we can confirm the recommendation of the IMPROVE guidelines and advise the monitoring of cardiovascular and respiratory parameters as continuously as possible from the induction until the discharge of anaesthesia (Percie du Sert et al., 2017). This includes surveillance of HR, blood pressure, respiratory rate and blood gases. Intubation and mechanical ventilation offer the possibility of immediate correction in case of deviations of the vital parameters. Invasive and non-invasive alternatives should be carefully selected, depending on factors such as laboratory conditions, experience of the experimenters, and experimental design. The maintenance of body temperature via a feedback system and strict control of the depth of anaesthesia is mandatory.

To control the correct induction of vessel occlusion, we consider bilateral CBF measurement in the intraluminal thread model to be an indispensable tool. It provides an immediate indication of the success of the intervention during the ongoing experiment and, thus, offers the possibility of a direct decision on whether to include or exclude the animals. In addition to the exclusion of undesired pathologies that falsify the results, there is a saving of time, work and money on the scientific side that should not be underestimated. The CBF measurement is also beneficial to animal welfare, as it avoids unnecessary suffering of the animals in the presence of undesirable pathologies, in accordance with the refinement of experimental procedures (Tannenbaum and Bennett, 2015).

In addition, behavioural tests may help to distinguish animals with only small lesions from those with larger infarction during the further course (Bernard et al., 2016; Metz, 2016). However, the specific tests have to be chosen carefully, and usually it is advisable to apply a battery of tests instead of a single one (Balkaya et al., 2018). There are several pitfalls, such as the misinterpretation of improvement through real functional recovery from (learned) compensation (Boltze et al., 2014). Computer-assisted and automated systems may offer an additional objective evaluation (Balkaya et al., 2018).

Furthermore, it should be mentioned that between the rat strains, and even within a single rat strain (dependent on the breeder), cerebrovascular anatomy, and therefore experimental results, may vary substantially (Fox et al., 1993; Oliff et al., 1995; Walberer et al., 2006). Thus, the experimental planning also has to be undertaken carefully, as even a change of breeder may influence the extent of ischemia.

We addressed a selected subtopic evaluating the quality assurance applying the tMCAO model. Of note, the model itself is debated controversially (Hossmann, 2012), but it is still one of the most common stroke models. Undesired results such as SAH are common, and a reliable detection of these pathologies is essential to minimize falsification of the experimental results and produce research as relevant as possible. Therefore, it is of utmost importance to make every effort to ensure the desired experimental result (ischemia).

Authors should discuss the results and how they can be interpreted in perspective of previous studies and of the working hypotheses. The findings and their implications should be discussed in the broadest context possible. Future research directions may also be highlighted.

In conclusion, we show the predictive value of periprocedural monitoring (CBF, MAP and HR) applying the intraluminal thread model to detect a successful induction of stroke reliably. It is an important issue to assure experimental quality and eliminate falsification of the experimental results by inclusion of undesired pathologies (such as SAH). Considering the poor translational power of preclinical stroke research, every effort has to be made to improve experimental methodology and transparency.

## MATERIALS AND METHODS

The experiments were performed at the Medical Faculty of the RWTH Aachen University, in accordance with German legislation governing animal studies (Tierschutzgesetz, Tierschutz-Versuchstierverordnung) and in accordance with the ARRIVE guidelines. The protocol (reference number 84-02.04.2013.A418) was approved by the State Agency for Nature, Environment and Consumer Protection (Landesamt für Natur, Umwelt und Verbraucherschutz Nordrhein-Westfalen).

This report was guided by the TRIPOD (Transparent Reporting of a multivariable prediction model for Individual Prognosis or Diagnosis) recommendations (Moons et al., 2015). In the framework of a randomized controlled animal study, we performed a retrospective analysis. Results of the underlying study have already partially been published (Liu et al., 2019).

### Animals

Male Wistar rats (Charles River, Sulzfeld, Germany) were housed for at least 1 week before surgery, with free access to food and water on a 12-h light/dark cycle, according to the Federation of European Laboratory Animal Science Associations health monitoring recommendations (Mahler Convenor et al., 2014).

### Induction of focal cerebral ischemia

Animals underwent 2 h of transient middle cerebral artery occlusion (tMCAO) using the intraluminal thread technique, as described previously (Liu et al., 2019). Briefly, general anaesthesia was applied with an intraperitoneal combination of 0.15 mg/kg body weight (BW) medetomidin (Domitor), 2 mg/kg BW midazolam (Midazolam-ratiopharm) and 0.005 mg/kg BW fentanyl (Rotexmedica). BW saline 10 ml/kg was administered every hour of anaesthesia as a subcutaneous depot for fluid substitution.

After loss of righting reflex, all animals were intubated endotracheally and mechanically ventilated using a small animal ventilator (Föhr Medical Instruments, RUS-1321-RA). Protective eye lubricant (Bepanthen) was administered to both eyes, body temperature was maintained at 37-37.5°C with a feedback-controlled warming plate (TCAT-2LV Controller, Physitemp) during the entire surgical intervention, and three electrocardiographic needle electrodes were placed for continuous HR monitoring. Surgical anaesthesia was monitored regularly by the pedal withdrawal reflex.

Bilateral laser Doppler flowmetry (moorVMS-LDF2, Moor Instruments) was used to measure CBF over both MCA-supplied territories during the entire experiment. A polyethylene catheter (Portex Polythene Tubing, SIMS) was inserted into the tail artery for blood pressure measurement.

After exposure of the distal left common carotid artery (CCA), a nylon filament with a silicone-coated tip (0.38±0.02 mm diameter) was introduced into the internal carotid artery (ICA) and advanced until resistance was felt and the lCBF measurement showed a simultaneous significant drop (t0). Two hours after MCAO, brain perfusion was restored by removal of the filament (t120). All parameters analyzed (HR, lCBF, rCBF, MAP and systolic/diastolic pressure) were continuously recorded with a data acquisition system (PowerLab, ADInstruments). Baseline measurement was taken 10-15 min after final setup and before surgical approach to the neck vessels; measured values were picked up every 5 min starting with the drop of CBF representing MCAO (t0) and ending 30 min after reperfusion (t150).

Animals were sacrificed either 3 h (3 h group), 7 days (7 days group) or 30 days after MCAO, according to the original study. Animals from the 30-day observation group were not included in the presented analysis. After euthanasia, the brains were immediately removed. The evaluation of the brains was carried out by an experienced neurosurgeon. The brains were carefully rinsed immediately after removal, examined under the microscope and photographed. With the help of subsequent TTC staining (3 h group) and Haematoxylin-Eosin (H&E) staining (for all animals, after 4% paraformaldehyde fixation, followed by microscopical assessment), a reliable diagnosis could be made depicting the ischemia and blood distribution.

### Outcome groups and experiments included

The analysis is based upon an original study (Deutsche Forschungsgemeinschaft, CO 799/9-1), including 203 tMCAO procedures (three observation times: 3 h, 7 days and 30 days). During the project, a considerable amount of procedures resulting in SAH or SDH were observed. In order to quantify the value of peri-interventional monitoring with respect to optimal tMCAO performance (resulting in ischemia), the present analysis was initiated. We did not include experiments from the 30 d observation group, as pathologies such as SAH are no more detectable at this time. Thus, we limited ourselves to animals (observation times: 3 h or 7 days) with a clear diagnosis based on macroscopic and microscopic examination.

Out of the initial data pool of 203 tMCAO experiments, 16 animals were selected to represent the ‘ideal’ group (outcome group I; ischemia in the territory supplied by the middle cerebral artery; observation times: 3 h, *n*=3; 7 days, *n*=13), whereas all animals with ‘undesired’ results detected after euthanasia (such as additional SDH and/or SAH; *n*=48; observation time: 3 h) formed outcome group II. Outcome group I was defined by the following inclusion criteria: macroscopic and microscopic confirmation of MCA-ischemia; exclusion of undesired pathologies (such as SAH or SDH) via macroscopic inspection and microscopic evaluation; and full peri-interventional monitoring (bilateral CBF measurement, blood pressure, heart frequency and temperature control) applied. Outcome group II was defined accordingly: confirmation of initially undesired pathology (such as SAH or SDH, which may be observed in addition to MCA-ischemia or independent from MCA-ischemia); and full peri-interventional monitoring (bilateral CBF measurement, blood pressure, heart frequency and temperature control) applied. Details of the animals are shown in Fig. 1. Only animals with a clear diagnosis based on macroscopic and microscopic examination, and corresponding documentation of vital parameters, were chosen.

One animal (SAH and MCA-ischemia) was euthanized before the end of the observation period because of apnea, but all collected data were analyzed. Another animal (SAH) was euthanized due to a bleeding complication during the further experimental course, but baseline and t0 values were saved. Furthermore, for four animals only incomplete monitoring data, not covering the entire procedure (2 h or less after MCA-occlusion), were available.

### Tissue preparation

After the initial photo documentation, the brains were either TTC-stained (every second slice, 3 h group) or immediately fixed in 4% paraformaldehyde. Then, the fixed sections were cut (2 mm) and embedded. From the start of each block, a 2-μm section was harvested for H&E staining and the infarct volume measurement taken.

#### Infarct volume measurement

Sections were stained with routine H&E, then visualized and photographed with an Axiovert 200M microscope (ZEISS; 10× objective). The images were analyzed using ZEN software (ZEISS). The sections were analyzed for confirmation of ischemia, identification of undesired pathologies, and measurement of infarct volume (outcome group I). The infarct volume was assessed using an indirect method: the volume of the non-lesioned volume of the ischemic hemisphere was subtracted from the total volume of the contralateral hemisphere. Afterwards, the volume was normalized to the volume of the contralateral hemisphere (same section).

### Statistical analysis methods

Data are presented as mean±s.d. Correlation between continuous variables was determined by Pearson's correlation coefficient (95% c.i.). We have captured the course of the lCBF through two different variants: the difference between mean lCBF before (t0 to t120) and the mean after occlusion (t120 to t150) (Δ_1_lCBF); and the difference between t0 and t120 (Δ_3_lCBF). The course of the rCBF were also captured by two variants: the difference between rCBF at baseline and at t0 (Δ_2_rCBF); and the mean of all time points (t0 to t150) (mean_rCBF). All single predictors were analyzed by univariate logistic regression with respect to experimental success.

Multiple logistic regression (PROC LOGISTIC) was used to investigate whether the specific variables (BL_HR; BL_MAP; Δ_1_lCBF, Δ_2_rCBF) predict the occurrence of unwanted pathologies (SAH, SDH or a mixed type). The prognostic performance is described by the ROC and corresponding AUC, including confidence interval. Here, the AUC corresponds to the c-index. For each model we reported *R*^2^, Brier Score and Akaike information criterion. We used leave-one-out cross validation imputed in PROC LOGISTIC as internal validation, and compared results using a corresponding Mann–Whitney U-Test.

If single values in the time course of CBF were missing, the summary measures for each rat were calculated for the available cases. Further missing values were reported. We assessed a 5% significance level for each model. Statistical analyses were performed using SAS Software (version 9.4, SAS Institute). Graphs were created using R Software (R Development Core Team, 2018) and GraphPad Prism (version 8.3.0).

## Supplementary Material

Supplementary information
