# Supplementary material for: Predicting experimental success: a retrospective case-control study using the rat intraluminal thread model of stroke
Source: Dis Model Mech. 2020 Dec 29;13(12):dmm044651. doi: 10.1242/dmm.044651 (PMC7790196; doi:10.1242/dmm.044651)
Supplement: Supplementary information [file dmm-13-044651-s1.pdf]

| predictor variables | outcome group I (n = 16) | | | outcome group II (n = 48) | | |
|---|---|---|---|---|---|---|
| | n | mean ± SD | median [Q1 - Q3] | n | mean ± SD | median [Q1 - Q3] |
| BL_HR | 16 | 263.27 ± 76.25 | 251.95 [232.8 - 290.72] | 48 | 198.03 ± 42.77 | 202.76 [176.89 - 221.6] |
| BL_DIAS | 16 | 87.61 ± 43.7 | 96 [68.96 - 127.57] | 48 | 138.81 ± 38.08 | 133.74 [118.83 - 154.85] |
| BL_SYS | 16 | 95.86 ± 46.4 | 104.29 [73.07 - 134.32] | 48 | 154.98 ± 42.59 | 149.01 [132.16 - 178.18] |
| BL_MAP | 16 | 90.36 ± 44.53 | 98.77 [71.01 - 129.82] | 48 | 144.2 ± 38.8 | 140.41 [121.66 - 162.83] |
| $\Delta_1$lCBF | 15 | 40.5 ± 37.4 | 26.26 [16.23 - 43.56] | 44 | -2.81 ± 15.02 | -4.43 [ -7.9 - 1.21] |
| $\Delta_2$rCBF | 15 | 2.71 ± 10.59 | 4.42 [ -2.43 - 8.27] | 45 | 54.86 ± 26.93 | 58.23 [32.76 - 79.49] |
| $\Delta_3$lCBF | 15 | -75.46 ± 49.39 | -55.94 [ -89.58 - -42.34] | 44 | -23.65 ± 47.31 | -11.99 [ -32.53 - -2.6] |
| mean_rCBF | 15 | 99.63 ± 16.43 | 101.52 [88.3 - 112.71] | 45 | 75.31 ± 39.1 | 66.9 [52.21 - 82.8] |
| BL_weight | 16 | 349.31 ± 36.73 | 347 [320 - 365] | 48 | 363.65 ± 42.76 | 356.5 [333 - 385.5] |

**Table S1** Description of all possible predictors. Baseline heart rate (BL_HR) in bpm; baseline diastolic pressure (BL_DIAS) in mmHg; baseline systolic pressure (BL_SYS) in mmHg; baseline mean arterial pressure (BL_MAP) in mmHg; difference between mean left cerebral blood flow (CBF) before (t0 to < t120) and the mean after occlusion (t120 to t150) ($\Delta_1$lCBF); difference between right CBF at baseline and at t0 ($\Delta_2$rCBF); difference between time point t0 and t120 of left CBF ($\Delta_3$lCBF); the mean of all time points of left CBF (t0 to t150) (mean_rCBF); initial weight in gram (BL_weight); standard deviation (SD); interquartile range (Q1-Q3)

| single predictor | n | beta coefficient of predictors | | | performance | | | multivariable model |
|---|---|---|---|---|---|---|---|---|
| | | log (OR) | SE | p-value | AUC | $R^2$ | Brier Score | |
| BL_SYS | 64 | 0.034 | 0.011 | 0.0017 | 0.83 | 0.25 | 0.13 | excluded |
| BL_DIAS | 64 | 0.038 | 0.012 | 0.0011 | 0.84 | 0.26 | 0.13 | excluded |
| BL_MAP | 64 | 0.037 | 0.012 | 0.0013 | 0.84 | 0.26 | 0.13 | included |
| BL_HR | 64 | -0.028 | 0.009 | 0.0028 | 0.83 | 0.23 | 0.13 | included |
| $\Delta_1$lCBF | 59 | 0.021 | 0.008 | 0.0086 | 0.89 | 0.16 | 0.15 | included |
| $\Delta_2$rCBF | 60 | 0.193 | 0.078 | 0.0132 | 0.97 | 0.52 | 0.07 | included |
| $\Delta_3$lCBF | 59 | 0.021 | 0.008 | 0.0086 | 0.95 | 0.42 | 0.09 | excluded |
| mean_rCBF | 60 | -0.018 | 0.009 | 0.0442 | 0.81 | 0.08 | 0.18 | excluded |
| BL_weight | 64 | 0.009 | 0.008 | 0.2348 | 0.59 | 0.02 | 0.18 | excluded |

**Table S2** Results of univariate logistic regression. Baseline heart rate (BL_HR); baseline diastolic pressure (BL_DIAS); baseline systolic pressure (BL_SYS); baseline mean arterial pressure (BL_MAP); difference between mean left cerebral blood flow (CBF) before (t0 to < t120) and the mean after occlusion (t120 to t150) ($\Delta_1$lCBF); difference between right CBF at baseline and at t0 ($\Delta_2$rCBF); difference between time point t0 and t120 of left CBF ($\Delta_3$lCBF); the mean of all time points of left CBF (t0 to t150) (mean_rCBF); initial weight (BL_weight); logarithm of estimated odds ratio (log(OR)); standard error (SE); area under the curve (AUC); R-squared ($R^2$); Brier score

| number of predictors | included predictors | n | beta coefficients of predictors | | | | | | | | | | model fitting information (AIC) | results of cross validation | | |
|---|---|---|---|---|---|---|---|---|---|---|---|---|---|---|---|---|
| | | | Intercept | | BL_HR | | BL_MAP | | $\Delta_1$lCBF | | $\Delta_2$rCBF | | | AUC CV | 95%-CI | Mann-Whitney-U |
| | | | log (OR) | SE | log (OR) | SE | log (OR) | SE | log (OR) | SE | log (OR) | SE | | | | |
| 1 | BL_HR | 64 | 7.3924 | 2.1666 | -0.0278 | 0.0093 | - | | - | | - | | 59.05 | 0.8 | (0.65,0.96) | 0.012 |
| | BL_MAP | 64 | -3.3585 | 1.3924 | - | | 0.037 | 0.0115 | - | | - | | 56.95 | 0.8 | (0.68,0.93) | 0.0013 |
| | $\Delta_1$lCBF | 59 | 2.3845 | 0.5953 | - | | - | | -0.1183 | 0.0339 | - | | 38.59 | 0.94 | (0.88,0.99) | 0.1005 |
| | $\Delta_2$rCBF | 60 | -2.1559 | 0.9173 | - | | - | | - | | 0.1932 | 0.078 | 27.89 | 0.95 | (0.9,1.00) | 0.1397 |
| 2 | BL_HR and BL_MAP | 64 | 1.3039 | 2.0886 | -0.0265 | 0.0096 | 0.047 | 0.0153 | - | | - | | 47.27 | 0.85 | (0.74,0.97) | 0.0341 |
| | BL_HR and $\Delta_1$lCBF | 59 | 10.7438 | 4.2256 | -0.0381 | 0.0177 | - | | -0.0963 | 0.034 | - | | 33.85 | 0.94 | (0.88,1.00) | 0.1205 |
| | BL_MAP and $\Delta_1$lCBF | 59 | -1.7308 | 1.6319 | - | | 0.0326 | 0.0133 | -0.1233 | 0.0409 | - | | 32.14 | 0.95 | (0.9,1.00) | 0.0531 |
| | BL_MAP and $\Delta_2$rCBF | 60 | -18.361 | 10.739 | - | | 0.1108 | 0.065 | - | | 0.294 | 0.1531 | 16.32 | 0.98 | (0.95,1.00) | 0.2214 |
| | BL_HR and $\Delta_2$rCBF | 60 | 2.5931 | 2.3293 | -0.0231 | 0.0118 | - | | - | | 0.2447 | 0.1113 | 24.37 | 0.96 | (0.92,1.00) | 0.1016 |
| | $\Delta_1$lCBF and $\Delta_2$rCBF | 59 | -1.3935 | 2.2317 | - | | - | | -0.2552 | 0.1286 | 0.2743 | 0.1807 | 13.51 | 0.98 | (0.96,1.00) | 0.1814 |
| 3 | BL_HR, BL_MAP and $\Delta_1$lCBF | 59 | 4.7206 | 5.0304 | -0.0424 | 0.0211 | 0.0558 | 0.0257 | -0.1117 | 0.0414 | - | | 26.24 | 0.96 | (0.91,1.00) | 0.115 |
| | BL_HR, BL_MAP and $\Delta_2$rCBF | 60 | -7.3086 | 10.368 | -0.0313 | 0.0282 | 0.0846 | 0.055 | - | | 0.2696 | 0.1384 | 16.72 | 0.98 | (0.96,1.00) | 0.1613 |
| | BL_HR, $\Delta_1$lCBF and $\Delta_2$rCBF | 59 | 39.0673 | 36.031 | -0.1995 | 0.1714 | - | | -0.662 | 0.4946 | 0.779 | 0.5899 | 8.97* | 0.98 | (0.94,1.00) | 0.2258 |
| | BL_MAP, $\Delta_1$lCBF and $\Delta_2$rCBF | 59 | -24.4921 | 30.512 | - | | 0.1593 | 0.2029 | -0.4358 | 0.5334 | 0.3298 | 0.3393 | 11.66 | 0.97 | (0.92,1.00) | 0.2383 |
| 4 | BL_HR, BL_MAP, $\Delta_1$lCBF and $\Delta_2$rCBF | 59 | 5.3979 | 77.764 | -0.1165 | 0.1533 | 0.1214 | 0.4413 | -0.569 | 0.5119 | 0.4553 | 0.4206 | 10.26* | 0.92 | (0.83,1.00) | 0.0638 |

**Table S3** Results of all multivariable logistic regression models. Baseline heart rate (BL_HR); baseline diastolic pressure (BL_DIAS); baseline systolic pressure (BL_SYS); baseline mean arterial pressure (BL_MAP); difference between mean left cerebral blood flow (CBF) before (t0 to < t120) and the mean after occlusion (t120 to t150) ($\Delta_1$lCBF); difference between right CBF at baseline and at t0 ($\Delta_2$rCBF); logarithm of estimated odds ratio (log(OR)); standard error (SE); area under the curve cross validation (AUC CV); Akaike information criterion (AIC); corresponding 95% confidence interval (95%-CI); p-values of Mann-Whitney-U-test (Mann-Whitney-U)

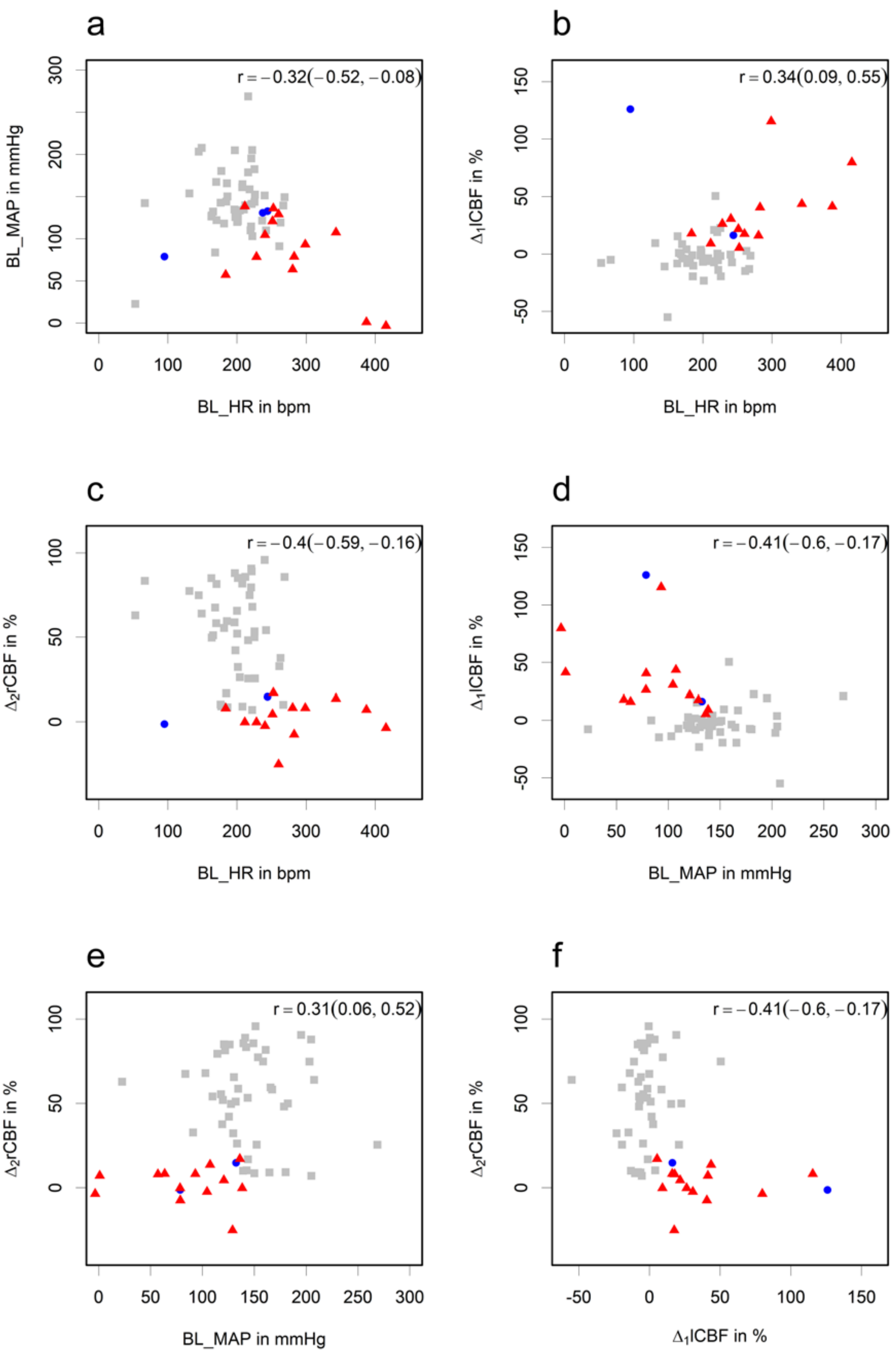


**Figure S1.** Correlation of **a** BL_MAP and BL_HR (n = 64) **b** $\Delta_1$lCBF and BL_HR (n = 59) **c** $\Delta_2$rCBF and BL_HR (n = 60) **d** $\Delta_1$lCBF and BL_MAP (n = 59) **e** $\Delta_2$rCBF and BL_MAP (n = 60) and **f** $\Delta_2$rCBF and $\Delta_1$lCBF (n = 59).

Disease Models & Mechanisms • Supplementary information

Baseline heart rate (BL_HR) in bpm; baseline mean arterial pressure (BL_MAP) in mmHg; difference between mean left cerebral blood flow before (t0 to < t120) and the mean after occlusion (t120 to t150) ($\Delta_1$lCBF) in %; difference between right cerebral blood flow at baseline and at t0 ($\Delta_2$rCBF) in %; data of animals sacrificed after 3 hours (outcome group I) are expressed as blue circles; data of animals sacrificed after 7 days (outcome group I) are expressed as red triangles; data of animals sacrificed after 3 hours (outcome group II) are expressed as grey squares

*The maximum likelihood estimate does not exist because of complete separation [1]. The information of the included predictors allow to separate completely outcome group I from outcome group II in this data set. Here, focus is on prediction not on specific estimate of the model. We only want to show the additional benefit of considering all possible predictors.

1. Albert A, Anderson JA. On the existence of maximun likelihood estimates in logistic regression models. *Biometrika*. 1984;71:1-10
